# SpeedCAP: An Efficient Method for Estimating Neural Activation Patterns Using Electrically Evoked Compound Action-Potentials in Cochlear Implant Users

**DOI:** 10.1097/AUD.0000000000001305

**Published:** 2022-12-08

**Authors:** Charlotte Garcia, John M. Deeks, Tobias Goehring, Daniele Borsetto, Manohar Bance, Robert P. Carlyon

**Affiliations:** 1 Cambridge Hearing Group, Medical Research Council Cognition and Brain Sciences Unit, University of Cambridge, Cambridge, United Kingdom; 2 Cambridge Hearing Group, Cambridge Universities Hospitals Foundation Trust, University of Cambridge, Cambridge, United Kingdom.

**Keywords:** Cochlear implant, ECAP, Neural activation patterns, Current spread, Neural health, Optimization

## Abstract

Electrically evoked compound action-potentials (ECAPs) can be recorded using the electrodes in a cochlear implant (CI) and represent the synchronous responses of the electrically stimulated auditory nerve. ECAPs can be obtained using a forward-masking method that measures the neural response to a probe and masker electrode separately and in combination. The panoramic ECAP (PECAP) analyses measured ECAPs obtained using multiple combinations of masker and probe electrodes and uses a nonlinear optimization algorithm to estimate current spread from each electrode and neural health along the cochlea. However, the measurement of ECAPs from multiple combinations of electrodes is too time consuming for use in clinics. Here, we propose and evaluate SpeedCAP, a speedy method for obtaining the PECAP measurements that minimizes recording time by exploiting redundancies between multiple ECAP measures.

**Design:**

In the first study, 11 users of Cochlear Ltd. CIs took part. ECAPs were recorded using the forward-masking artifact-cancelation technique at the most comfortable loudness level (MCL) for every combination of masker and probe electrodes for all active electrodes in the users’ MAPs, as per the standard PECAP recording paradigm. The same current levels and recording parameters were then used to collect ECAPs in the same users with the SpeedCAP method. The ECAP amplitudes were then compared between the two conditions, as were the corresponding estimates of neural health and current spread calculated using the PECAP method previously described by Garcia et al. The second study measured SpeedCAP intraoperatively in 8 CI patients and with all maskers and probes presented at the same current level to assess feasibility. ECAPs for the subset of conditions where the masker and probe were presented on the same electrode were compared with those obtained using the slower approach leveraged by the standard clinical software.

**Results:**

Data collection time was reduced from ≈45 to ≈8 minutes. There were no significant differences between normalized root mean squared error (RMSE) repeatability metrics for post-operative PECAP and SpeedCAP data, nor for the RMSEs calculated *between* PECAP and SpeedCAP data. The comparison achieved 80% power to detect effect sizes down to 8.2% RMSE. When between-participant differences were removed, both the neural-health (r = 0.73) and current-spread (r = 0.65) estimates were significantly correlated (*p* < 0.0001, df = 218) between SpeedCAP and PECAP conditions across all electrodes, and showed RMSE errors of 12.7 ± 4.7% and 16.8 ± 8.8%, respectively (with the ± margins representing 95% confidence intervals). Valid ECAPs were obtained in all patients in the second study, demonstrating intraoperative feasibility of SpeedCAP. No significant differences in RMSEs were detectable between post- and intra-operative ECAP measurements, with the comparison achieving 80% power to detect effect sizes down to 13.3% RMSE.

**Conclusions:**

The improved efficiency of SpeedCAP provides time savings facilitating multi-electrode ECAP recordings in routine clinical practice. SpeedCAP data collection is sufficiently quick to record intraoperatively, and adds no more than 8.2% error to the ECAP amplitudes. Such measurements could thereafter be submitted to models such as PECAP to provide patient-specific patterns of neural activation to inform programming of clinical MAPs and identify causes of poor performance at the electrode-nerve interface of CI users. The speed and accuracy of these measurements also opens up a wide range of additional research questions to be addressed.

## INTRODUCTION

Cochlear implants (CIs) are neuroprosthetic devices that provide auditory sensations to individuals with severe-to-profound hearing loss by electrical stimulation of the auditory nerve. Many CI users understand speech well in quiet situations, but there is a lot of variability in speech performance among CI users, particularly in situations with background noise (Friesen et al. 2001; Firszt et al. 2004). As a result, a large volume of research has been invested in investigating measurements that might predict CI speech performance. Many models have been developed for this prediction, incorporating a myriad of behavioral and objective measurements with varying degrees of reported accuracy (Kim et al. 2010; Lazard et al. 2012; Scheperle & Abbas 2015; Kim et al. 2017; Schvartz-Leyzac & Pfingst 2018; Carlyon & Goehring 2021). However, these measurements are often summary metrics for CI user populations and do not provide detailed estimates of an individual user’s unique pattern of neural excitation at the electrode-nerve interface. This latter information may be important for optimizing efficacy of CIs and improving speech perception (Long et al. 2014; Pfingst et al. 2015). Obtaining multiple measurements along the length of the electrode array for a particular CI user is sufficiently time consuming to be a barrier to use in clinical settings, especially if utilizing behavioral techniques. Objective measurements can be faster and more automated, but they may require additional hardware. For example, electrically evoked auditory brainstem responses (EABRs) reflect synchronous physiological responses to electrical stimulation from various stages of the auditory pathway from the peripheral auditory nerve to the inferior colliculus (Hall III 1992), but require additional electrodes to be placed on a patient’s scalp for measurements (Brown et al. 1994).

Electrically evoked compound action-potentials (ECAPs) are the most practical objective measurements, as they require no additional hardware beyond the computer interface, and leverage the CI electrodes already inserted inside the cochlea both to stimulate and record. ECAPs measure the synchronous peripheral neural response to a current pulse delivered by an electrode in a CI, recorded with a nearby intra-cochlear electrode (Charlet de Sauvage et al. 1983; Brown et al. 1990). ECAPs are measured routinely in clinical practice, for example, to estimate threshold (T) and comfort (C) levels, although their relevance for predicting behavioral assessments of T and C levels has been questioned (de Vos et al. 2018). Several studies have further suggested that they can be used to estimate various aspects of neural excitation patterns such as spiral-ganglion neuron survival (Prado-Guitierrez et al. 2006; Ramekers et al. 2014). These have also been used in more complex models to estimate neural excitation patterns in individual users. Cosentino et al. (2015) described the first version of the panoramic ECAP (PECAP) method that measured ECAPs with the forward-masking artifact-cancelation technique for all combinations of masker and probe electrodes at equal loudness levels. The resultant measurement matrix M (with rows and columns containing ECAP P2-N1 amplitudes corresponding to probe and masker electrodes respectively) was then processed using an algorithm that estimated underlying excitation patterns for each electrode. Biesheuvel et al. (2016) described a model using similar ECAP data at equal current levels that estimate excitation density profiles by treating them as convolutions for different electrodes. Garcia et al. (2021) presented a refinement of the PECAP algorithm that processed M using a nonlinear optimization algorithm in order to estimate variation in current spread and neural health along the electrode array in individual CI users. This revised version of the PECAP algorithm, in particular, shows promise for clinical use as it was able to identify localized areas of simulated reduced neural responsiveness (“dead” regions) using its neural-health estimate, suggesting that the estimate does, in fact, estimate neural health accurately (Garcia et al. 2021). While the analysis techniques described in Cosentino et al., Biesheuvel et al., and Garcia et al. can be conducted in a matter of seconds once the relevant ECAP data have been obtained, the PECAP method and other methods of this kind require the measurement of many ECAPs. Individual ECAPs take only a few seconds to record, but it takes approximately 45 minutes to collect ECAPs for all combinations of masker and probe electrodes in the Cochlear Ltd. (Sydney, Australia) clinical software, Custom Sound EP. This limits implementation in routine clinical practice, as time is already severely constrained (and costly) in the clinic.

Here, we present a new method, called SpeedCAP, for efficiently measuring multiple ECAPs to speed up the process of collecting the data required to implement the PECAP algorithm. SpeedCAP exploits various redundancies present in the ECAP-recording process when using the forward-masking artifact-cancelation technique (Abbas et al. 1999) with many combinations of masker and probe electrodes. Using the SpeedCAP method, it takes only about 8 minutes to record the measurement matrix M required for the PECAP algorithm compared with approximately 45 minutes when using the standard method. The previously-standard ≈ 45-minute method for recording the M matrix will be referred to as SlowCAP throughout this article. Note that “SpeedCAP” and “SlowCAP” refer to ECAP-recording procedures, whereas “PECAP” refers here to the analysis conducted on the ECAP data that are used to populate the M matrix in order to extrapolate patient-specific estimates of current spread and neural health along the cochlea. The SpeedCAP method is evaluated by determining how well it can replicate the ECAP amplitudes obtained with the SlowCAP method using root mean squared error (RMSE) metrics averaged across the M matrices and the current-spread and neural-health estimates from the PECAP algorithm described in Garcia et al. (2021).

While the method has been applied in this study to the ECAPs required for the PECAP algorithm using Cochlear Ltd. devices only, the techniques required to speed up the data collection process can also be applied to other ECAP analyses such as spread of excitation functions (Cohen et al. 2003), and be used in principal with other CI manufacturers’ devices. The SpeedCAP method opens up new possibilities to assess the electrode-nerve interface of individual CI users with the PECAP method in clinical environments, to provide better insights into CI stimulation patterns for optimizing fitting and improving patient outcomes.

## METHODS

### SpeedCAP Techniques and Assumptions

Here, we provide a description of the techniques used to optimize the data acquisition process for the PECAP method. The standard PECAP measurement matrix M described in Garcia et al. (2021) is an ***n*** × ***n*** matrix that contains ECAP amplitudes obtained with the forward-masking artifact-cancelation technique from every combination of probe and masker electrode, where ***n*** is the number of electrodes switched on in the patient’s MAP.

SpeedCAP combines three ways of reducing the data collection time. First, only a part of the PECAP measurement matrix M is recorded. Second, communication time between the testing computer and the implant processor is reduced by recording multiple ECAPs at once and transmitting them from the implant processor in batches. Third, SpeedCAP skips frames in the forward-masking artifact-cancelation technique that are repeated when recording multiple ECAPs, so as to prioritize recording of nonredundant frames and to reduce data traffic.

**Fig. 1.**
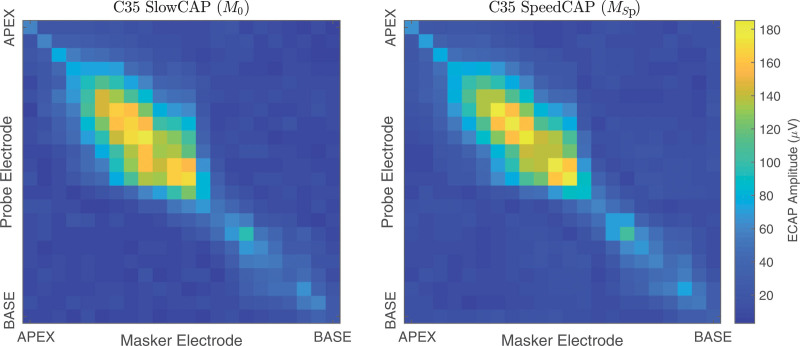
(left) An example of a full PECAP matrix, $M_{0}$, from participant C35. Each cell represents the amplitude of an ECAP waveform in μV, one for each of every possible combination of masker and probe electrodes. It can be seen that the patterns of ECAP amplitudes are predominantly mirrored across the diagonal of the matrix. (right) An example of the corresponding SpeedCAP,$M_{Sp}$, matrix from the same participant. It can be seen that the $M_{0}$ and $M_{Sp}$ matrices show similar ECAP patterns. ECAPs, electrically evoked compound action-potentials; PECAP, panoramic ECAP.

The first time-saving technique is the recording of only a part of M based on the assumption that two ECAPs are theoretically and computationally equivalent when using the forward-masking artifact-cancelation technique if the masker and probe electrodes are swapped—that is, M is symmetric across its diagonal. Although this may not be completely true, we and others have modeled the ECAP in terms of the overlap between the masker and probe excitation patterns (Cosentino et al. 2015; Biesheuvel et al. 2016; Garcia et al. 2021) and our previous recordings suggested that asymmetries in M are rather small. Indeed, PECAP initially processes the M to force it to become symmetric before processing by the nonlinear optimization algorithm. This assumption will be evaluated below and a “raw” measurement matrix (one that has not already been made symmetrical) is illustrated in Figure 1 (left). For the case where the Mmatrix is recorded using all 22 electrodes and 50 sweeps are presented for each ECAP at a rate of 80 Hz, it will take ≈ 45 minutes to record all 22 × 22 = 444 waveforms. It is important to note at this point that the recorded voltage waveforms in Cochlear Ltd.’s telemetry system are averaged across all sweeps before delivery to the computer, and therefore the individual responses to each sweep are not nominally available. While in principle it should only take about 20 minutes to record 444 50-sweep waveforms at a rate of 80 Hz, it takes longer than this due to the communication time between the implant processor and the computer (≳1 second in each direction for each ECAP). We can reduce this to approximately 25 minutes in the first instance simply by skipping half of the ECAPs for which we have assumed equivalence due to symmetry of ***M***. This would mean recording ECAPs for the cases where the masker and probe are presented on the same electrode (referred to as the diagonal), as well as where the masker electrode is basal to the probe electrode (or vice versa), resulting in a total of 253 ECAP waveforms.

**Fig. 2.**
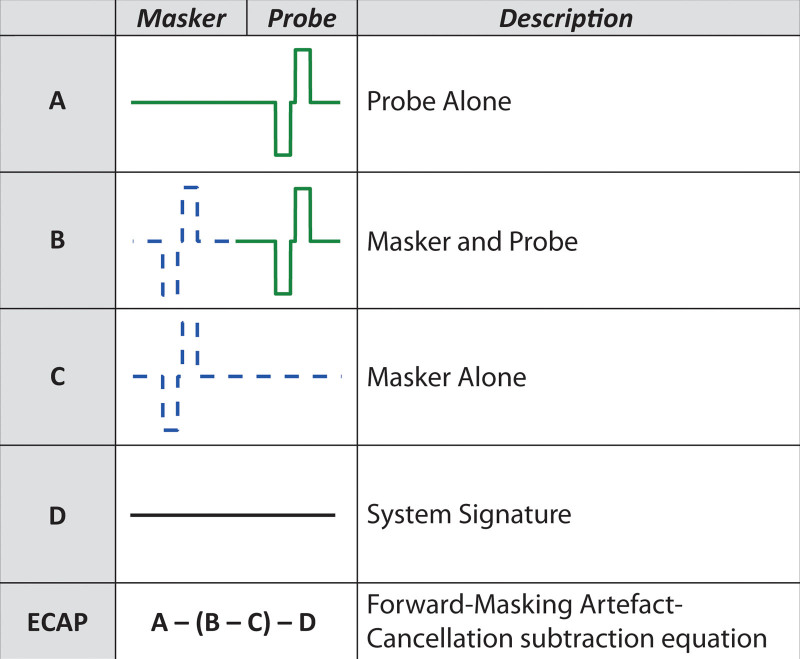
Graphical representation of the four stimulation frames required to extract the neural response from the stimulus artifacts using the forward-masking artifact-cancelation technique for ECAPs. ECAPs, electrically evoked compound action-potentials.

The second technique reduces the overhead communication time between the testing computer and the implant processor. With the current clinical software used to record ECAPs with Cochlear Ltd. devices (Custom Sound EP), it takes approximately 1 second for the software to send the neural response telemetry (NRT) commands to the implant processor, and approximately one additional second to deliver the recorded data from the implant processor back to the computer after recording. Using the NIC2 research software provided by Cochlear Ltd., it is possible to collect the four frames required to extract an ECAP using the forward-masking artifact-cancelation technique four at a time. In other words, the A, B, C, and D frames depicted in Figure 2 can be collected from the implant processor for 4 different ECAPs in a single batch with no additional time added between frames. As the transmission time of data between the processor and the computer appear to be independent of the number of frames transmitted, the overhead communication time required to collect ECAP amplitudes can be reduced by 75% using this technique alone. For the 253 ECAPs in the reduced-sized ***M*** matrix, the ≈ 8.5 minutes of overhead communication time in the ≈ 25-minute data acquisition sequence can be reduced to ≈ 2 minutes, saving a further ≈ 6.5 minutes.

**Fig. 3.**
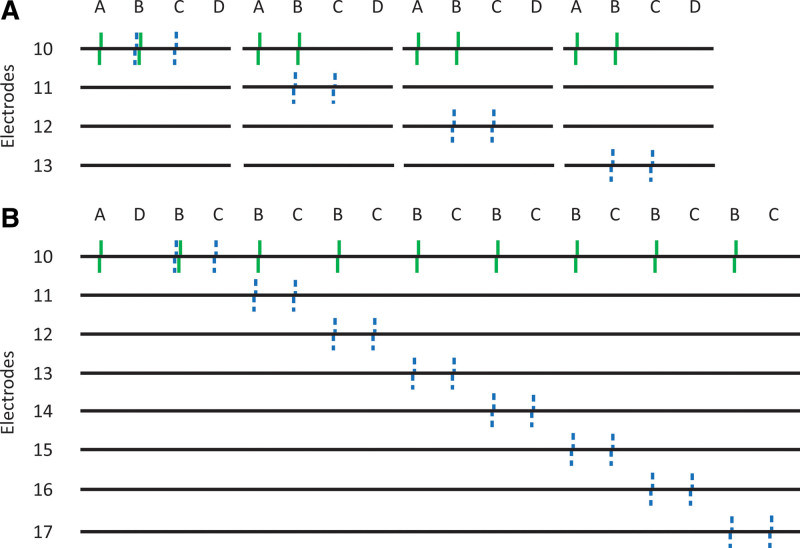
A, example pulse-train sequence for recording 4 ECAPs using the standard forward-masking artifact-cancelation technique. Each black line on the y-axis indicates a different electrode on which the biphasic pulses are delivered, and the frame types as described in Figure 2 are labeled on the x-axis, with probe pulses indicated by solid green lines and masker pulses indicated by dashed blue lines. B, example pulse-train sequence for recording 8 ECAPs using the forward-masking artifact-cancelation technique and recycling the A and D frames. ECAPs, electrically evoked compound action-potentials.

Finally, the forward-masking artifact-cancelation technique has various redundant frames when recording ECAPs from many combinations of masker and probe electrodes. For example, when the probe remains on the same electrode and the masker is moved along the array, both the probe-alone frame (A in Figure 2) and the system-signature frame (D in Figure 2) are repeated with exactly the same recording parameters for each ECAP. In order to optimize recording time, SpeedCAP only records these frames once and re-uses them to extract ECAP waveforms, where the masker is on different electrodes. Combining this technique with the batch-downloading time-saving technique described above, it is possible to record enough frames to calculate ECAP waveforms for 8 combinations of masker and probe electrodes at one time. This is depicted in Figure 3B, and the standard forward-masking pulse-train sequence is depicted in Figure 3A for reference. This number is limited to 8 ECAPs because the size of the NIC2 buffer only supports transfer of 19 frames of data from the implant processor to the computer at one time. One instance of this 8-ECAP recording sequence takes approximately 14.5 seconds when presenting 50 sweeps.

Combining the three techniques described, the proposed SpeedCAP recording matrix takes only ≈ 8 minutes to record (253 50-sweep ECAPs), instead of the ≈ 45 minutes required to record the standard PECAP recording matrix (444 50-sweep ECAPs). While it is possible to employ these techniques to achieve an 8-minute SpeedCAP recording method, the precise mathematics of the time-saving contributions of each technique when implemented simultaneously cannot be further elaborated upon without being privy to more of the internal software of the NIC2 system than is afforded to a user. It is possible that these techniques and others could be leveraged more optimally with additional knowledge of the proprietary software, and SpeedCAP could be recorded in even less than 8 minutes. Figure 1 (right) shows an example of a SpeedCAP matrix recorded using the techniques described above and is from the same patient as the SlowCAP ***M*** matrix shown (Figure 1, left).

### Data Collection: Study 1

#### Participants

Eleven users of Cochlear Ltd. CI devices were recruited. Informed consent was obtained from each participant in accordance with the International Research Code of Ethics (1990) with care to abide by international standards of ethics (i.e., the Bulletin of the Pan American Health Organization, 24, 604-621) at the MRC Cognition & Brain Sciences Unit in Cambridge, United Kingdom. Permission to conduct the study was granted by the National Research Ethics committee for the East of England (Ref no: A08225), and all participants provided their written consent to participate. They were reimbursed for their travel costs and were compensated for volunteering their time. The participants had a range of etiologies and device types, and averaged 61 years of age (standard deviation = 13.4 years). Demographic and recording-parameter information is listed in Table 1.

**TABLE 1.** Participant information for the postoperative study (study 1).

ID	Implanted Ear	Age	Etiology	Device	Electrodes Assessed	ECAP recording Gain (dB)/Delay (µs)	ECAP Pulse Width (µs)	SlowCAP Recording Electrode Side	SpeedCAP Recording Electrode Side
**C09**	Right	69	Hereditary	CI24RE	3–21	50/98	42	Basal	Basal
**C13**	Right	57	Maternal rubella and ear infection	CI522	1–22	50/122	25	Apical	Basal*
**C19L**	Left	66	Exposure to loud sounds	CI512	1–20	50/73	42	Apical	Basal*
**C26**	Right	57	Hereditary	CI522	1–22	60/122	25	Basal	Basal
**C27**	Right	71	Progressive sensorineural	CI512	1–21	60/98	25	Apical	Apical
**C30**	Right	71	Measles	CI512	1–22	50/122	25	Basal	Basal
**C31**	Right	27	Hereditary	CI622	3–22	50/73	37	Apical	Apical
**C32**	Left	66	Meningitis	CI612	1–22	50/73	37	Apical	Apical
**C33**	Right	56	Superficial siderosis	CI622	3–22	50/98	50	Apical	Apical
**C34**	Left	70	Progressive	CI24RE	2–22	50/73	25	Apical	Apical
**C35**	Right	73	Meniere’s disease	CI622	1–22	50/98	37	Apical	Apical

* Recording paradigm and recording electrode side confounded, and only 1 repeat of SpeedCAP available.

#### Panoramic ECAP Measurements

The standard PECAP matrix (***M***) was measured for all combinations of masker and probe electrodes active in the participant’s clinical MAP at most comfortable levels (MCL). These data were collected as per the methods described in detail in Garcia et al. (2021). Custom software programmed using the NIC2 platform from Cochlear Ltd. allowed for the acquisition of four 12-sweep sets of the four frames (A, B, C, and D) required to extract an ECAP waveform with the forward-masking artifact-cancelation technique, and allowed for post-acquisition averaging that amounted to a total of 48 sweeps per masker-probe electrode combination. Although the clinical standard is to record 50-sweep ECAP waveforms, the sub-sampling of measurements into four sets of 12-sweep ECAPs allowed us to calculate the repeatability metrics presented in the Results section. ECAP amplitudes were calculated from each of these 48-sweep waveforms and assembled into ***n*** × ***n*** matrices, where ***n*** indicates the number of electrodes from which ECAPs were recorded for an individual participant. These data (in ***M*** format), obtained with the method described in Garcia et al. (2021), will be referred to as SlowCAP and $M_{0}$ throughout the remainder of this article for brevity. The subscript “0” is chosen for consistency with the nomenclature used in Garcia et al. (2021) and indicates the original method of recording ECAPs.

On the same day, SpeedCAP measurements were then additionally performed for each participant with the same recording parameters as used for the SlowCAP data (i.e., gain, delay, pulse width, recording electrode side, and current levels) for each electrode. The only exceptions to this were participants C13 and C19L for whom the recording electrode for the SpeedCAP measurements was placed 2 electrodes basal to the probe electrode, whereas for the SlowCAP measurements it was placed 2 electrodes apical to the probe electrode (see Table 1). SpeedCAP was recorded twice for each participant, both times with 50 sweeps for each ECAP frame, except for participants C13 and C19L from whom only one SpeedCAP recording was obtained due to time restrictions and interruptions during data collection due in part to the COVID-19 pandemic. The sub-sampling of ECAP waveforms into four sets of 12 sweeps was not possible for the SpeedCAP data collection procedure due to the limited size of the NIC2 buffer used to transfer data from the implant processor the computer. The SpeedCAP data will be abbreviated as $M_{Sp}$ throughout the remainder of this article.

Before the main data collection, recording parameters such as current level required for MCL, gain, and delay settings were determined using the Custom Sound EP Software. The main data were obtained using custom software programmed in Python (version 2.4.4, Python Software Foundation, USA) through the PyCharm IDE interface (Community Edition 2016.1.5, JetBrains, Czech Republic) using the NIC2 research platform (Cochlear Ltd., Sydney, Australia). MATLAB R2018a (Mathworks, Natick, Massachusetts, USA) was used to visualize and analyze data using the panoramic ECAP Method.

### Data Collection: Study 2

#### Participants

Eight severe-to-profoundly deaf patients scheduled to receive cochlear implants from Cochlear Ltd. were recruited before undergoing surgery. Participants were informed of the nature of the study and provided written consent to participate before undergoing their implant surgery. Informed consent was obtained in accordance with the International Research Code of Ethics (1990) with care to abide by international standards of ethics (i.e., Bulletin of the Pan American Health Organization, 24, 604-621). Ethical approval for the study was obtained from HRA and Health and Care Research Wales (HCRW) through University Hospitals NHS Foundations Trust and the University of Cambridge (IRAS ID: 285894, Ref No: A095798, REC Reference: 20/EM/0263). Three participants also took part in Study 1 (C32, C33, C35). Demographic and recording-parameter information for these patients is included in Table 2.

**TABLE 2.** Participant information for the intraoperative study (study 2).

ID	Implanted Ear	Age	Etiology	Device	ECAP Recording Gain (dB)/ Delay (µs)	ECAP Pulse Width (µs)	Current Level(CUs)	SpeedCAP Recording Electrode Side
C32	Left	66	Meningitis	CI612	50/73	37	180	Apical
C33	Right	56	Superficial siderosis	CI622	50/98	50	220	Apical
S4	Right	21	Congenital, progressive	CI622	40/98	50	235	Apical
S5	Right	76	Congenital, progressive	CI622	50/98	25	240	Apical
C35	Right	73	Meniere’s disease (L) and infection (R)	CI622	50/98	37	180	Apical
S8	Left	20	Congenital, unknown	CI622	50/73	37	210	Apical
S9	Left	63	Progressive, unknown	CI522	40/98	50	210	Apical
S10	Left	71	Bilateral Meniere’s disease	CI622	40/98	37	225	Apical

#### Intraoperative ECAP Measurements

After the cochlear implant electrode array was inserted into the cochlea by the surgeon, recording parameters required to elicit a valid ECAP response intraoperatively were determined using Custom Sound EP software. The pulse width, current level, gain, and delay settings required were noted (shown in Table 2). Custom software developed in Python (version 2.4.4, Python Software Foundation, USA) and compiled into a stand-alone executable program was then used to record ECAPs using the parameters determined in Custom Sound EP with equal current level for all 22 electrodes. The first recording consisted of batches of four ECAP waveforms—each an average of 12 sweeps, resulting in 48 sweeps in total—along the diagonal of the PECAP matrix to confirm that valid ECAPs were recorded using the chosen parameters. Once this was complete, the program recorded SpeedCAP in the same manner as described in the previous section but with equal current levels for all electrodes across the implant array instead of equal loudness levels as used for the postoperative ECAPs. Recording SpeedCAP at equal loudness levels was not possible intraoperatively as the patient was under general anesthesia for the surgical procedure. An advantage was that the current levels used, although necessarily limited by safety considerations, were not limited by the loudness that an awake patient would find comfortable. The entire intraoperative ECAP measurement procedure took approximately 10–15 minutes and was completed at the end of the surgery, before awakening the patient from general anesthesia.

### Panoramic ECAP (PECAP) Algorithm

While a key portion of this manuscript investigates the ability of the SpeedCAP method to record ECAP waveforms that result in the same peak-peak (P2-N1) amplitudes in the Mmatrices that are obtained by the SlowCAP method, the results section will also submit these $M_{0}$ and $M_{Sp}$ matrices to the panoramic ECAP (PECAP) algorithm to estimate patient-specific patterns of current spread and neural health and compare the estimates obtained using the two different methods. Therefore, it is useful to briefly explain the PECAP algorithm and how it uses the M data to arrive at these estimates.

**Fig. 4.**
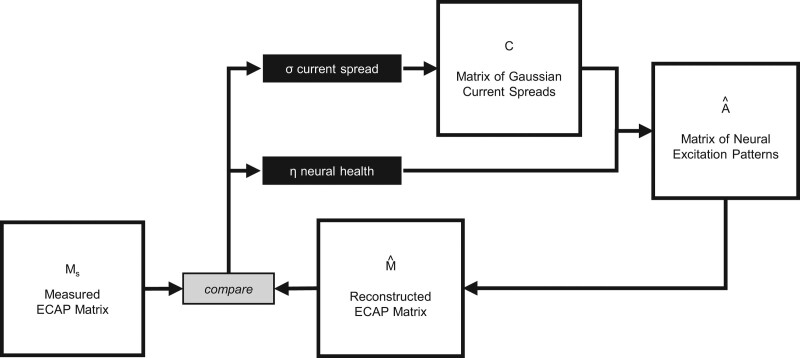
Schematic for the PECAP algorithm. The optimization algorithm based on sequential quadratic programming adjusts the values in the σ and η vectors, reconstructs M, and updates σ and ηiteratively in order to minimize the RMSE between $M_{0}$ and M. RMSE, root mean squared error; PECAP, panoramic ECAP.

The PECAP algorithm described by Garcia et al. (2021) assumes that each measured ECAP in the M matrices is determined by the overlap of the excitation patterns produced by the corresponding masker and probe. It also assumes that these excitation patterns are influenced both by the spread of electrical current from each electrode, and the synchronized peripheral responsiveness of the neurons to electrical stimulation (termed “neural health” for brevity). It formalizes these two contributory aspects of the neural excitation patterns by representing the current spread with a Gaussian curve of standard deviation σ centered on each electrode (each of these making up a row in a matrix C), and using a normalizing vector to represent relative responsiveness of neurons close to each electrode (referred to as η). Each row of C is then multiplied by the vector η to form the underlying neural excitation pattern matrix A. From A, it can be inferred what the measurement matrix M should be, but as the algorithm is an ill-defined inverse problem, hard limits, and smoothness constraints are applied before minimizing the difference between the recreated M matrix, and the observed one (for this article, either $M_{0}$ or $M_{Sp}$) using a nonlinear optimization algorithm based on sequential quadratic programming. The schematic for the structure of the PECAP algorithm is displayed in Figure 4. The key thing to remember for the purposes of this article is that the input data to the algorithm is the recorded matrix of ECAP amplitudes (either $M_{0}$ or $M_{Sp}$, examples of which are displayed in Figure 1), and the outputs are the two vectors describing current spread for each electrode (σ) and neural health along the array (ƞ). (The current spread vector simply contains the standard deviations of the Gaussian curves in each row of the Cmatrix.) It is these two output vectors estimated using the two different methods of recording ECAPs (SlowCAP vs SpeedCAP) that are compared in the analysis of study 1.

## RESULTS

### Study 1: Postoperative Comparison Between SlowCAP and SpeedCAP

#### ECAP Measurement Assessment

The SlowCAP and SpeedCAP matrices ($M_{0}$ and $M_{Sp}$, respectively) were evaluated for repeatability before comparison. Our hypothesis was that if using the SpeedCAP method was *not* equivalent to the SlowCAP method, then the errors shown by the comparison of SpeedCAP to SlowCAP would be greater than when comparing the repeats of the same method. To compensate for participant-wise differences in ECAP amplitudes, the root mean squared error (RMSE) between repeat measurements of $M_{0}$ and $M_{Sp}$ were normalized by the maximum ECAP amplitude in $M_{0}$ and $M_{Sp}$ for each participant, respectively. This allowed us to compare the RMSE values between participants and meant that the RMSE metrics were reported in percentage of the maximum ECAP amplitude instead of micro-Volts (µV).

Equation (1) was used to calculate the normalized within-condition RMSE $\varepsilon_{S}$ for SpeedCAP:


$$\begin{array}{l} \varepsilon_{S}=\frac{1}{\max\left(M_{Sp1},M_{Sp2}\right)}\cdot \sqrt{\bar{{\left(M_{Sp1}-M_{Sp2}\right)}^{2}}} \end{array}$$ (1)

where $M_{Sp1}$ is the first SpeedCAP measurement taken and $M_{Sp2}$ is the second. The $M_{Sp}$ matrices are made up of full n x n matrices where n is the number of electrodes assessed for each participant, but as indicated by the x-bar above the squared difference between $M_{Sp1}$ and $M_{Sp2}$, the cell-wise RMSE is averaged across the whole matrix to give rise to a single value, $\varepsilon_{Sp}$. $M_{p,m}$ represents the ECAP amplitude in the cell of the $M_{Sp}$ matrix that corresponds to the condition where the probe is on electrode p and the masker is on electrode m. The full $M_{Sp}$ matrix is completed with $M_{p,m}$ amplitudes copied across the diagonal to the $M_{m,p}$ locations as the latter are not recorded. This is done for the error calculation because the data must be in this format before submission to the PECAP Algorithm.

Equation (2) was used to calculate the normalized within-condition RMSE $\varepsilon_{0}$ for SlowCAP:


$$\begin{array}{l} \begin{array}{l} \varepsilon_{0}=\frac{1}{\sqrt{2}\cdot \max\left(M_{0a},M_{0b}\right)}\cdot \sqrt{\overset{}{\bar{{\left(M_{0a}-M_{0b}\right)}^{2}}}\;} \end{array} \end{array}$$ (2)

where $M_{0a}$ is the matrix of ECAP amplitudes calculated using sweeps 1–24 to form waveforms for each ECAP, and $M_{0b}$ uses sweeps 25–48. Since halving the number of sweeps increases the measurement noise in the ECAP waveform by a factor of $\sqrt{2}$ (Underraga et al. 2012; Stronks et al. 2019), we incorporated this into the calculation so that $\varepsilon_{0}$ represents the noise in the 48-sweep waveform ECAP amplitudes that make up $M_{0}$. To enable a direct comparison between $\varepsilon_{Sp}$ and $\varepsilon_{0}$, both $M_{0a}$ and $M_{0b}$ are constructed symmetrically in the same manner as described above for $M_{S}$ matrices, including only the $M_{p,m}$ cells with the same masker and probe conditions as the $M_{Sp}$ matrices for each participant. In some cases, this consisted of $M_{p,m}$ conditions with the masker located basally to the probe electrode, and in others the masker was located apically to the probe electrode.

SlowCAP ($M_{0}$) and SpeedCAP ($M_{Sp}$) were then compared by calculating the normalized between-condition RMSE using equation (3):


$$\begin{array}{l} \begin{array}{l} \varepsilon_{0,S}=\frac{1}{\max\left(M_{0},M_{Sp}\right)}\cdot \sqrt{\overset{}{\bar{{\left(M_{0}-M_{Sp}\right)}^{2}}}\;} \end{array} \end{array}$$ (3)

where $M_{0}$ consists only of $M_{p,m}$ cells with the same masker and probe conditions as $M_{Sp}$, calculated from the full 48-sweep waveforms.

Our assumption that recording only part of the M matrix does not introduce significant error is additionally evaluated below using equation (4):


$$\begin{array}{l} \begin{array}{l} \varepsilon_{sym}=\frac{1}{\max\left(M_{0,asym},M_{0,sym}\right)}\cdot \sqrt{\overset{}{\bar{{\left(M_{0,asym}-M_{0,sym}\right)}^{2}}}\;} \end{array} \end{array}$$ (4)

where $M_{0,asym}$ is the SlowCAP ($M_{0}$) matrix using all 48-sweep ECAP waveforms recorded from every combination of masker and probe electrode, and $M_{0,sym}$ is the same SlowCAP ($M_{0}$) made symmetric across the diagonal according to equation (5):


$$\begin{array}{l} \begin{array}{l} M_{0,sym}=\frac{M_{0,asym}+M_{0,asym}^{\prime }}{2} \end{array} \end{array}$$ (5)

where $M_{0,asym}^{\prime }$ is the transposition of$M_{0,asym}$. $M_{0,sym}$ is the format of the data nominally submitted to the panoramic ECAP algorithm (Garcia et al. 2021) and therefore $\varepsilon_{sym}$ is of interest when considering the practical implications of implementing SpeedCAP in addition to assessing the symmetry assumption.

**Fig. 5.**
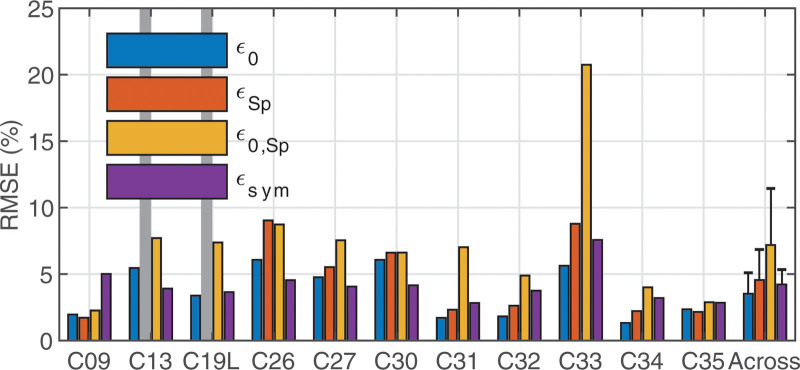
RMSEs for repeatability of SlowCAP ($\varepsilon_{0}$) and SpeedCAP ($\varepsilon_{Sp}$) data, as well as for the comparison of the two ECAP recording paradigms ($\varepsilon_{0,Sp}$) and the symmetry assumption ($\varepsilon_{sym}$). The right-most column shows the across-participant means for each of the four error metrics for each of the 9 participants included in the ANOVA (this excludes C13 and C19L for whom these metrics could not be calculated, as indicated by the vertical gray bars), none of which are statistically significant from each other. The error bars indicate 95% confidence intervals around the mean. RMSE, root mean squared error; PECAP, panoramic ECAP.

The $\varepsilon_{0}$, $\varepsilon_{Sp}$, $\varepsilon_{0,Sp}$, and $\varepsilon_{sym}$ values for each participant are shown in Table 3, as well as graphically depicted in Figure 5. Since only one $M_{Sp}$ was available for participants C13 and C19L, it was not possible to calculate $\varepsilon_{Sp}$ for them and as such they were excluded from further analysis. Our question of whether SpeedCAP and SlowCAP are significantly different from each other can be tested by comparing the repeatability metrics ($\varepsilon_{0}$ and $\varepsilon_{Sp}$) to the comparison one ($\varepsilon_{0,Sp}$). A one-way analysis of variance (ANOVA) was conducted using MATLAB (2018a) between the four ε metrics for the remaining 9 participants. After correcting for multiple comparisons using the Bonferroni-Dunn method, neither $\varepsilon_{0}$, $\varepsilon_{Sp}$, $\varepsilon_{0,Sp}$, nor $\varepsilon_{sym}$ significantly differed from each other (*p* = 0.13, df = 3, F = 2.02), indicating that we cannot reject our null hypothesis.

**TABLE 3.** RMSEs for postoperative SlowCAP ($M_{0}$) and SpeedCAP ($M_{Sp}$), calculated as described in equations (1), (2), (3), and (4)

Participant	RMSE (%)			
	SlowCAP ($\varepsilon_{0}$)	SpeedCAP ($\varepsilon_{Sp}$)	Comparison ($\varepsilon_{0,Sp}$)	SlowCAP Symmetry($\varepsilon_{sym})$
C09	1.96	1.72	2.26	5.00
C13	5.46	n/a	7.71	3.91
C19L	3.38	n/a	7.37	3.64
C26	6.07	9.04	8.73	4.54
C27	4.76	5.52	7.54	4.06
C30	6.07	6.61	6.61	4.15
C31	1.71	2.32	7.02	2.82
C32	1.82	2.61	4.89	3.75
C33	5.63	8.78	20.75	7.56
C34	1.32	2.21	4.00	3.20
C35	2.35	2.15	2.87	2.84
Across	3.52 ± 1.60	4.55 ± 2.29	7.19 ± 4.25	4.12 ± 1.12

The “Across” metrics are the means of the RMSEs for all participants except C13 and C19L, ±95% confidence intervals. n/a = not applicable.

It should be noted, that the lack of evidence of an effect is not evidence for lack of an effect; the nonsignificant *p* value resulting from the ANOVA does not directly indicate that SpeedCAP and SlowCAP are equivalent to each other, just that there is no evidence that they are different from each other. To assess margins of equivalence, 95% confidence intervals were calculated for the mean difference in RMSE between each of the four ε metrics, resulting in six overall comparisons. The highest upper 95% confidence limit was 8.2% and resulted from the comparison between $\varepsilon_{0,Sp}$ and $\varepsilon_{0}$. This indicates that while the ANOVA showed no evidence for statistically significant differences between $\varepsilon_{0}$, $\varepsilon_{Sp}$, $\varepsilon_{0,Sp}$, nor $\varepsilon_{sym}$, the data suggests that in 95% of future cases, differences between any of the metrics would be smaller than 8.2%. Therefore, it can be concluded that replacing SlowCAP with the SpeedCAP method of measuring ECAPs introduces no more than 8.2% error in ECAP amplitudes.

#### Neural Activation Patterns Assessment

**Fig. 6.**
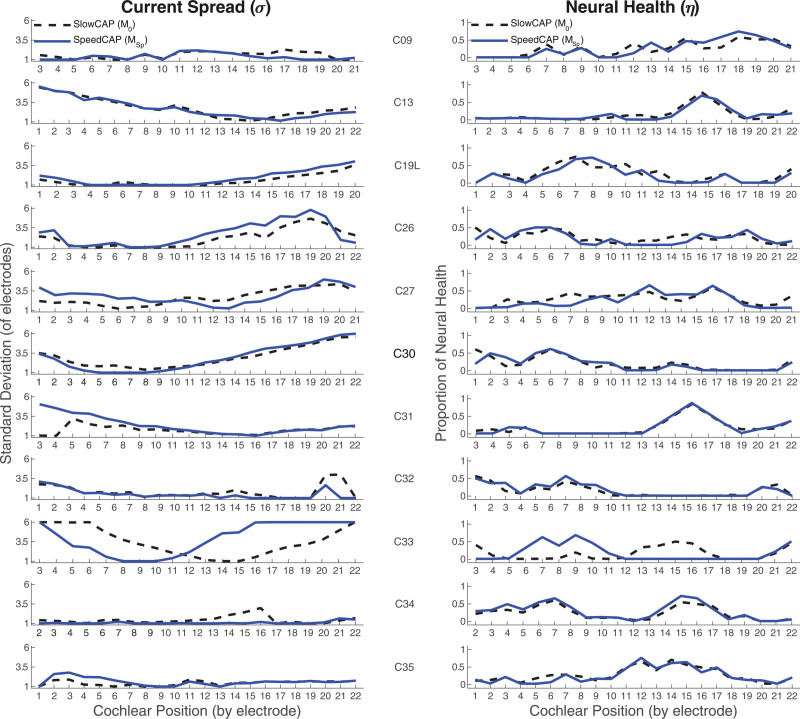
Current Spread (σ) and Neural Health (η) estimates from the PECAP algorithm using SlowCAP ($M_{0}$) measurements indicated by the dashed black lines, and SpeedCAP ($M_{Sp}$) measurements indicated by the solid blue lines. PECAP, panoramic ECAP.

SlowCAP ($M_{0}$) and SpeedCAP ($M_{Sp}$) data were then submitted to the PECAP algorithm described in Garcia et al. (2021). Note that for this analysis, $M_{0}$ is the symmetric version of SlowCAP ($M_{0,sym}$) obtained using equation (5) and that is nominally submitted to the PECAP algorithm. This section therefore evaluates the practical implications on the PECAP algorithm of replacing SlowCAP with the SpeedCAP method. Estimates of current spread and neural health along the cochlea were calculated for each participant using either $M_{0}$ (SlowCAP) or $M_{Sp}$ (SpeedCAP). The results of the PECAP algorithm are shown in Figure 6 for each of the 11 participants, with the dashed black lines representing the current-spread (σ) and neural-health (η) estimates when $M_{0}$ was submitted to the PECAP algorithm, and the solid blue lines representing the estimates when $M_{Sp}$ was submitted to the PECAP algorithm. With the exception of C33, the patterns of the current-spread and neural-health estimates generally agree between the two conditions.

**Fig. 7.**
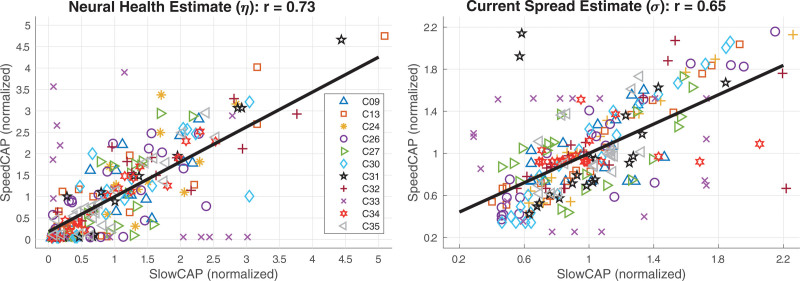
Across-electrode correlations for the (left) current spread (σ) and (right) neural health (ƞ) estimates from PECAP between the $M_{0}$ and $M_{Sp}$ data with between-participant differences removed. PECAP, panoramic ECAP.

Across-electrode, within-participant correlations between neural-health estimates for $M_{0}$ versus $M_{Sp}$ were significant in 10 of 11 cases with Pearson r Wvalues ranging from 0.59 to 0.97. There was only one case, participant C33, for whom there was no significant correlation for the neural-health estimate. Across-electrode, within-participant correlations between current-spread estimates for $M_{0}$ versus $M_{Sp}$ were significant in 8 of 11 cases with Pearson r values ranging from 0.51 to 0.96. There were no significant correlations for the current-spread estimates for participants C31, C33, and C34. Across all electrodes with between-participant differences removed, both the neural-health (r = 0.73, *p* < 0.0001, df = 218) and current-spread (r = 0.65, *p* < 0.0001, df = 218) estimates were significantly correlated between the SlowCAP and SpeedCAP conditions. Table 4 contains details of correlation coefficients and *p* values for all within- and across-participant comparisons, and Figure 7 shows the correlations for the neural-health and the current-spread estimates between $M_{0}$ versus $M_{Sp}$ across all electrodes.

**TABLE 4.** Correlation coefficients and *p* values for the current spread (σ) and neural health (ƞ) estimates from PECAP between the $M_{0}$ and $M_{Sp}$ data

Subject	Current Spread(σ)		Neural Health(η)	
	r value	*p*	r value	*p*
**C09**	**0.51**	0.026	**0.81**	***
**C13**	**0.96**	***	**0.91**	***
**C19L**	**0.96**	***	**0.83**	***
**C26**	**0.90**	***	**0.59**	0.004
**C27**	**0.55**	0.009	**0.70**	**
**C30**	**0.98**	***	**0.81**	***
**C31**	0.26	0.26	**0.98**	***
**C32**	**0.61**	0.003	**0.90**	***
**C33**	0.08	0.75	-0.15	0.52
**C34**	0.19	0.42	**0.96**	***
**C35**	**0.53**	0.011	**0.92**	***
**ALL**	**0.65**	***	**0.73**	***

Significant r values are shown in **bold**.

**p < 0.001, ***p < 0.0001.

**Fig. 8.**
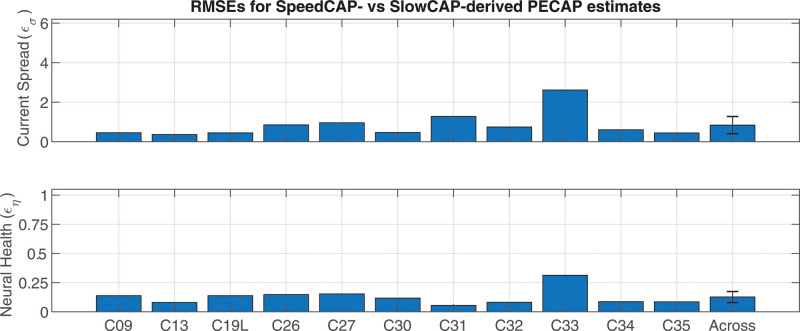
RMSEs for the across-electrode (top) current spread ($\varepsilon_{\sigma }$) and (bottom) neural health ($\varepsilon_{\eta }$) estimates from PECAP between the $M_{0}$ and $M_{Sp}$ data. The error bars represent the 95% confidence intervals around the across-participant $\varepsilon_{\sigma }$ and $\varepsilon_{\eta }$ means. RMSE = root mean squared error; PECAP = panoramic ECAP.

Error metrics (RMSEs) were also calculated between current-spread estimates derived from submitting the SlowCAP matrix ($M_{0}$) to the PECAP algorithm ($\sigma_{0}$) and those derived from submitting the SpeedCAP matrix ($M_{Sp}$) to the PECAP algorithm ($\sigma_{sp}$) using equation (6) below:


$$\begin{array}{l} \begin{array}{l} \varepsilon_{\sigma }=\sqrt{\bar{\overset{}{{\left(\sigma_{0}-\sigma_{sp}\right)}^{2}}\;}} \end{array} \end{array}\begin{array}{l} \begin{array}{l} \varepsilon_{\sigma }=\sqrt{\bar{\overset{}{{\left(\sigma_{0}-\sigma_{sp}\right)}^{2}}\;}} \end{array} \end{array}$$ (6)

where $\sigma_{0}$ is the current-spread estimate as a result of submitting the SlowCAP ($M_{0}$) matrix to the PECAP algorithm and $\sigma_{sp}$ is the current-spread estimate as a result of submitting the SpeedCAP ($M_{Sp}$) matrix to the PECAP algorithm. The same error metric was calculated for the neural-health estimate as described below in equation (7):


<tex−math><tex−math> (7)

where $\eta_{0}$ is the neural-health estimate as a result of submitting the SlowCAP ($M_{0}$) matrix to the PECAP algorithm and $\eta_{sp}$ is the neural-health estimate as a result of submitting the SpeedCAP ($M_{Sp}$) matrix to the PECAP algorithm. The individual $\varepsilon_{\sigma }$ and $\varepsilon_{\eta }$ metrics as well as the across-participant means are displayed in Figure 8. The across-participant error for the current-spread estimate calculated using SlowCAP versus SpeedCAP Mdata were 0.84 ± 0.44 standard deviations by electrode (95% confidence intervals). As the range of possible values for cells of σ is from 1 to 6 standard deviations by electrode in the PECAP algorithm, this represents 16.8 ± 8.8% of the total range of current-spread estimates. The across-participant error for the neural-health estimate using SlowCAP versus SpeedCAP M data were 0.13 ± 0.05 (95% confidence intervals), and as the range of possible values for cells of η is 0 to 1 in the PECAP algorithm representing relative proportion of neural responsiveness, this represents 12.7 ± 4.7% of the total range of neural-health estimates. A paired two-sided t-test revealed no significant difference between normalized values of $\varepsilon_{\sigma }$ and $\varepsilon_{\eta }$ (*p* = 0.16, t-stat = 1.52, df = 10, 95% CI = –0.02 to 0.10), providing no evidence that the error in estimating current-spread is different than the error in estimating neural-health between the two recording conditions. Note that participant C33 who showed no significant correlation as indicated in Table 4 is also an outlier in these calculations and presents the highest error values for both $\varepsilon_{\sigma }$ and $\varepsilon_{\eta }$ as shown in Figure 8, likely artificially inflating the across-participant error calculations.

**Fig. 9.**
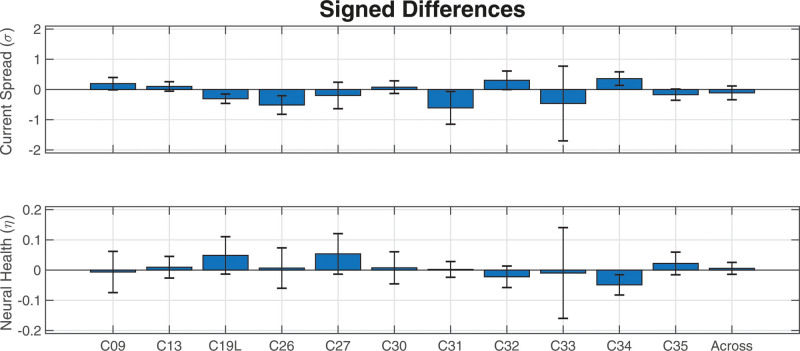
Mean signed differences for the (top) current spread (σ) and (bottom) neural health (ƞ) estimates from PECAP between the $M_{0}$ and $M_{Sp}$ data. The error bars represent the 95% confidence intervals around the means. For both metrics, positive values indicate $\sigma_{0}$ > $\sigma_{Sp}$ or ${}_{\eta_{0}}$ > $\eta_{Sp}$ where $\sigma_{0}$ and $\eta_{0}$ are the current spread and neural health estimates from the PECAP algorithm using SlowCAP ($M_{0}$) data, and $\sigma_{Sp}$ and $\eta_{Sp}$ are the current spread and neural health estimates from the PECAP algorithm using SpeedCAP ($M_{Sp}$) data. RMSE = root mean squared error; PECAP = panoramic ECAP.

In addition to the correlational and error analyses between the SlowCAP and SpeedCAP conditions, the average signed error was calculated for the current-spread (σ) and neural-health (ƞ) estimates to investigate whether there were trends of over- or underestimation when using SpeedCAP as opposed to SlowCAP data. The results can be seen in Figure 9. The across-participant mean signed differences were not statistically significant from 0 for current spread (two-sided t-test: *p* = 0.29, df = 1, F = 1.18) nor for neural health (two-sided t-test: *p* = 0.52, df = 1, F = 0.43), providing no evidence to support the hypothesis that SpeedCAP data either over- or under-estimated ECAP neural-activation estimates compared with SlowCAP data.

### Study 2: Intraoperative SpeedCAP Robustness

#### Diagonal ECAP Measurement Assessment

The limited time available during surgery made it impossible to collect multiple $M_{Sp}$ datasets intraoperatively. However, repeated measures of the ECAPs along the diagonal of the PECAP matrix could be collected. Therefore, it was possible to calculate $\varepsilon_{0}$ and $\varepsilon_{0,Sp}$ for the intraoperative data using only ECAPs from conditions where the probe and masker used the same electrode (the diagonal of the PECAP matrix). The intraoperative ECAP measurements along the diagonal will be referred to as $D_{0}$, and the diagonal of the intraoperative SpeedCAP data will be referred to as $D_{Sp}$. Due to the reduced number of ECAPs available to calculate the repeatability and reliability metrics for the intraoperative dataset, a subscript ε_*d*_ is used to indicate that only the diagonal and not the entirety of the PECAP matrix was included. Similarly to our hypothesis for study 1, the hypothesis here was that if using the SpeedCAP method was *not* equivalent to the SlowCAP method, then the error reflected by the comparison metric would be greater than for the repeatability metric.

The equation to calculate the error for the intraoperative SlowCAP diagonal ($D_{0}$) is displayed in equation (8):


$$\begin{array}{l} \begin{array}{l} \varepsilon_{d0}=\frac{1}{\sqrt{2}\cdot \max\left(D_{0a},D_{0b}\right)}\cdot \sqrt{\bar{\overset{}{{\left(D_{0a}-D_{0b}\right)}^{2}}\;}} \end{array} \end{array}\begin{array}{l} \begin{array}{l} \varepsilon_{d0}=\frac{1}{\sqrt{2}\cdot \max\left(D_{0a},D_{0b}\right)}\cdot \sqrt{\bar{\overset{}{{\left(D_{0a}-D_{0b}\right)}^{2}}\;}} \end{array} \end{array}$$ (8)

where $D_{0a}$ is a vector of ECAP amplitudes along the diagonal calculated using sweeps 1–24 of each ECAP, and $D_{0b}$ using sweeps 25–48 of each ECAP obtained using SlowCAP. Data were not available to calculate the $\varepsilon_{dSp}$ metric corresponding to the postoperative $\varepsilon_{Sp}$ (equation 1), but the equation for the between-condition RMSE for the intraoperative data using both $D_{0}$ and $D_{Sp}$ is displayed in equation (9):


$$\begin{array}{l} \begin{array}{l} \varepsilon_{d0,Sp}=\frac{1}{\max\left(D_{0},D_{Sp}\right)}\cdot \sqrt{\bar{\overset{}{{\left(D_{0}-D_{Sp}\right)}^{2}}\;}} \end{array} \end{array}\begin{array}{l} \begin{array}{l} \varepsilon_{d0,Sp}=\frac{1}{\max\left(D_{0},D_{Sp}\right)}\cdot \sqrt{\bar{\overset{}{{\left(D_{0}-D_{Sp}\right)}^{2}}\;}} \end{array} \end{array}$$ (9)

where $D_{0}$ and $D_{Sp}$ ECAP amplitudes are calculated using the full 48-sweep waveforms.

**Fig. 10.**
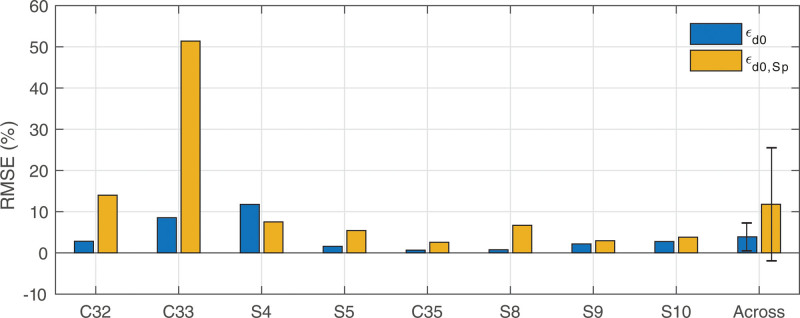
$\varepsilon_{d0}$ and $\varepsilon_{d0,Sp}$ for intraoperative datasets, calculated using the Diagonal of the ECAP matrices only. The right-most column shows the across-participant means for each of the two ε metrics. No significant difference was detectable between the two groups. The error bars indicate 95% confidence intervals around the mean.

The $\varepsilon_{d0}$ and $\varepsilon_{d0,Sp}$ values for each intraoperative participant are included in Table 5, as well as graphically depicted in Figure 10. Note that $\varepsilon_{d0,Sp}$ does not appear to be markedly higher than $\varepsilon_{d0}$ for any individual participants except for C33, who was also an outlier in the analysis presented from study 1. A paired-sample t-test was conducted using MATLAB (2018a) between the two metrics and did not reveal a significant difference (*p* = 0.17, t-stat = 1.52, df = 7, 95% CI = –4.46 to 20.29).

**TABLE 5.** $\varepsilon_{d0}$ and $\varepsilon_{d0,Sp}$ for intraoperative datasets, calculated using the diagonal of the ECAP matrices only ($D_{0}$ and $D_{Sp}$ for SlowCAP and SpeedCAP, respectively).

Participant	RMSE (%)	
	SlowCAP ($\varepsilon_{d0}$)	Comparison ($\varepsilon_{d0,Sp}$)
C32	2.82	14.00
C33	8.53	51.38
S4	11.76	7.52
S5	1.60	5.43
C35	0.66	2.59
S8	0.76	6.69
S9	2.17	2.95
S10	2.76	3.81
Across	3.88 ± 3.40	11.80 ± 13.72

The “Across” metrics are the means of the RMSEs for all participants, ±95% confidence intervals.

#### Postoperative versus Intraoperative ECAP Errors

A further analysis was done to compare the $\varepsilon_{0}$ and $\varepsilon_{0,Sp}$ for the postoperative datasets to the corresponding $\varepsilon_{d0}$ and $\varepsilon_{d0,Sp}$ for the intraoperative datasets. The motivation for this comparison was to determine whether reliability of the $D_{Sp}$ and $D_{0}$ data was significantly lower intraoperatively compared with the postoperative $M_{Sp}$ and $M_{0}$ data.

Neither of the comparable error metrics were significantly different from each other intra- versus postoperatively: a one-way ANOVA performed between $\varepsilon_{0}$ and $\varepsilon_{d0}$ resulted in a nonsignificant result (*p* = 0.89, df = 1, F = 0.02, n1 = 11, n2 = 8), and a second one-way ANOVA performed between $\varepsilon_{0,Sp}$ and $\varepsilon_{d0,Sp}$ also resulted in a nonsignificant result (*p* = 0.39, df = 1, F = 0.76, n1 = 11, n2 = 8).

However, as these were across-participant as opposed to within-participant analyses, these two comparisons had relatively low statistical power compared with the other statistical tests calculated in these studies. Using G*Power software (version 3.1.9.2, Aichach, Germany), it was determined that the data had 80% power to detect an effect size of a Cohen’s d equal to 1.2. Therefore, while no evidence was available to suggest that there were significant differences in the εmetrics intra- versus postoperatively, the data only had power to detect differences larger than 3.5% RMSEs for the $\varepsilon_{0}$ metrics and 13.3% RMSEs for the $\varepsilon_{0,Sp}$ metrics.

## DISCUSSION

### Robustness of SpeedCAP as an ECAP Recording Technique

The analysis of variance in the SpeedCAP recording technique in comparison to the standard ECAP recording paradigm for the panoramic ECAP measurement methods showed no evidence for a difference in repeatability of the ECAP amplitudes calculated from SpeedCAP data compared with the standard recording paradigm. Our estimate of 95% confidence limits suggests that for this group of participants the reliability and repeatability of ECAP amplitudes using the SpeedCAP method is no more than ≈ 8% different from the standard recording paradigm. The analysis also showed no evidence for a difference between the repeatability metrics for either SpeedCAP ($\varepsilon_{Sp}$) or SlowCAP ($\varepsilon_{0}$) and the comparison metric ($\varepsilon_{0,Sp}$). Hence, our analyses imply no more than ≈ 8% error in ECAP amplitudes is incorporated when replacing the standard ECAP recording paradigm with the faster SpeedCAP method. In addition to this, the analysis showed no evidence for a difference between either of the repeatability metrics ($\varepsilon_{Sp}$ and $\varepsilon_{0}$) nor the comparison metrics $\varepsilon_{0,Sp}$ and the error incorporated when the standard SlowCAP data is made symmetrical for submission to the panoramic ECAP algorithm ($\varepsilon_{sym}$). Therefore, there is no evidence that replacing SlowCAP with SpeedCAP introduces more error to the ECAP amplitudes than this standard symmetricalization of $M_{0}$ already implemented by Garcia et al. (2021) for estimation of neural health and current spread using the PECAP algorithm.

There was one participant in the dataset for whom the $\varepsilon_{0,S}$ metric visually appeared to be an outlier. For this participant, C33, the repeatability metrics for both SpeedCAP ($\varepsilon_{Sp}$) and SlowCAP ($\varepsilon_{0}$) did *not* appear to be outliers, suggesting that significant, reliable differences in the ECAP amplitudes *would* be present between SpeedCAP and SlowCAP. It is possible that this difference could be due to C33’s particular etiology; superficial siderosis is a rare condition in which hemosiderin is deposited on the pial surface of the brain. This condition presents commonly with atrophy of neural tissue and could mean that the peripheral auditory nerves take longer to recover from stimulation than with normal hearing or other hearing-related conditions (Kumar 2007). The primary difference in stimulation patterns between SlowCAP and SpeedCAP is that SpeedCAP stimulates more areas of neural tissue along the length of the cochlea in quicker succession than in SlowCAP. The stimulation rate of 80 pulses per second (pps) for both SlowCAP and SpeedCAP allows the stimulated areas of neural tissue to recover from a refractory state between successive ECAP recording frames under normal circumstances, but in the presence of siderosis, there may be other longer-term temporal properties of the neural tissue that elongate refractory periods or mean they may need longer to fully recover between successive stimulation for other reasons. There are other conditions, such as cochlear nerve deficiency, where the pulse rate is often slowed down to 15–20 pps from the standard 80 pps for ECAP recording to allow the neural tissue more time to recover between stimulation frames due to longer absolute refractory periods (He et al. 2016; Xu et al. 2020; Zhan et al. 2021). In such cases and with other hearing pathologies that elongate the temporal recovery properties of the auditory nerve, SpeedCAP may not be practically equivalent to standard recording techniques for ECAPs. However, when slowing down the rate is necessary for a particular patient group, measuring SpeedCAP at that rate may result in more similar ECAP amplitudes to the standard (SlowCAP) method, and will certainly still be more efficient.

### Use of SpeedCAP for Estimating Neural Activation Patterns

High and significant correlations as well as relatively low RMSE metrics for the neural-health (ƞ) and current-spread (σ) estimates between the SpeedCAP ($M_{Sp}$) and SlowCAP ($M_{0}$) conditions suggest that similar neural excitation patterns can be estimated using the panoramic ECAP method described by Garcia et al (2021) when using the SpeedCAP method for collecting these data instead of the standard recording paradigm. More of the individual participant correlations were significant between conditions for the neural-health estimate (10 of 11 participants) than for the current-spread estimate (8 of 11 participants), suggesting that employing the faster data collection procedure may slightly sacrifice accuracy in current-spread estimates. However, there was no evidence that the errors were greater for the current-spread estimate ($\varepsilon_{\sigma }$= 16.8 ± 8.8% RMSE) than for the neural-health estimate ($\varepsilon_{}$ = 12.7 ± 4.7% RMSE) between conditions, suggesting this may not be the case. One participant showed no significant correlation for the neural-health nor current-spread estimates between the SpeedCAP and SlowCAP methods as well as high RMSE values, providing an exception to our general conclusion that SpeedCAP is a suitable method for measuring neural activation patterns in CI patients. However, it can be seen that the patterns of neural health and current spread appear to be quite similar for SpeedCAP and SlowCAP for the other participants, suggesting that in most cases, SpeedCAP data may be used to estimate neural excitation patterns in CI users using the Garcia et al. (2021) method. However, we have reported the margins of error as opposed to directly assessing the equivalence because it is not yet clear what an effect size of interest is for either the PECAP method’s estimate of current spread or neural health.

### Feasibility of Intraoperative SpeedCAP

The second study showed that it was feasible to record viable ECAPs using SpeedCAP intraoperatively, after the electrode array was in place in the cochlea, before awakening the patient from general anesthesia. No evidence was found for a difference between the repeatability of the intraoperative SlowCAP measures of the diagonal ($\varepsilon_{d0}$) and the comparison metric between these data and the diagonal of the intraoperative SpeedCAP measures ($\varepsilon_{d0,Sp}$), consistent with the postoperative analysis in the first study. The data also show that if a difference did exist it would be below ≈ 20.3% RMSE, as this is the smallest effect size the comparison had 80% power to detect. This suggests that the conclusion that no practical difference exists between SlowCAP and SpeedCAP measures holds true for intraoperative measurements in addition to postoperative ones.

There was also no evidence that recording SlowCAP or SpeedCAP intraoperatively led to any difference in repeatability or comparison metrics compared with postoperative data, further supporting reliability and feasibility of intraoperative SpeedCAP measurements. However, future work investigating intra- versus postoperative SpeedCAP data is underway and will investigate any potential differences with greater statistical power in order to assess feasibility of estimating neural activation patterns using intraoperative SpeedCAP data and transform the panoramic ECAP method into a fully objective tool.

### Clinical Implications

The time savings involved with the SpeedCAP method for recording ECAPs compared with SlowCAP has substantial implications for the clinical viability of the panoramic ECAP method. In many scientific fields, speeding up the process of collecting data is a “nice-to-have” aspect of the research methods. However, time is of the essence for the clinical application of new techniques, and resources are both costly and limited in the clinic and particularly in the operating theater. Therefore, long measurements such as the ≈ 45 minutes that were previously required to collect data for the panoramic ECAP method are prohibitively long to be a viable diagnostic tool that could be used in routine clinical practice. The 8-minute SpeedCAP data collection method is sufficiently quick such that these data can now be collected in the clinic without requiring additional resources, and can be collected intraoperatively without adding significant time to the surgical procedures. This transforms the panoramic ECAP method from a research-exclusive tool into a method practical to use in clinical environments, and opens up new possibilities for assessing the CI electrode-nerve interface and for applying precision medicine approaches such as PECAP to cochlear implant healthcare. This claim should not be over-exaggerated; however, the effectiveness of using the panoramic ECAP method to improve CI speech perception has not yet been sufficiently evaluated so as to render it a method that is recommended for widespread clinical implementation at this stage, and further work in this area is needed.

In addition to transforming PECAP into a clinically viable tool, similar principles to SpeedCAP for obtaining quicker recordings of otherwise uncollectable data due to time-constraints could be applied to other measurement procedures. For example, any research study that utilizes intraoperative ECAP measures (e.g., Söderqvist et al. 2021) could benefit from collecting all spread of excitation functions of interest instead of being restricted in their data collection by limited time in the operating theater. Of course, this is not limited to the operating theater; even in research settings where multiple hours are available for data collection from cochlear implant research volunteers, collecting ECAP data in a more efficient way allows for more conditions to be evaluated without extending research time.

## CONCLUSION

SpeedCAP, a new, more efficient method for measuring multiple ECAPs using the forward-masking artifact-cancelation technique, has been presented and validated against the standard ECAP recording paradigm. This method takes only ≈ 8 minutes to record in Cochlear Ltd. CI devices instead of ≈ 45 minutes, and transforms the panoramic ECAP method for estimating patient-specific neural activation patterns from a research laboratory method into a potentially clinically viable tool. While further work is required to establish this transition, practically speaking, SpeedCAP appears to be broadly equivalent to the standard recording paradigm for calculating ECAP amplitudes and estimating patient-specific neural activation patterns. The quality of these data recorded intraoperatively was similar to postoperative data. As well as providing methods that open up more possibilities in clinical environments to assess neural responsiveness using ECAPs and ask more detailed research questions, this is a step forward in development of clinically viable diagnostic tools for precision medicine in cochlear implant healthcare, and may lead to better insights into stimulation patterns for optimizing fitting and CI patient outcomes.

## ACKNOWLEDGMENTS

The W. D. Armstrong Trust Fund & The Cambridge Trust supported corresponding author C.G. Authors T.G. and R.P.C. were supported by awards MR/T03095X/1 and MCUU0005/3, respectively, from the U.K. Medical Research Council.
